# Sensitivity to permethrin in a *Dermacentor reticulatus* population from eastern Poland in laboratory study

**DOI:** 10.1186/1756-3305-7-18

**Published:** 2014-01-10

**Authors:** Alicja Buczek, Katarzyna Bartosik, Paweł Kuczyński

**Affiliations:** 1 Chair and Department of Biology and Parasitology, Medical University of Lublin, Radziwiłłowska11 St. 20-080, Lublin, Poland; 2 Orthopedics and Rehabilitation Clinic, Independent Public Clinical Hospital nr 4 in Lublin, Lublin, Poland

**Keywords:** *Dermacentor reticulatus*, Tick control, Permethrin, Reproduction, Egg development

## Abstract

**Background:**

The action of chemical compounds on the Palaearctic tick *D. reticulatus* (Fabricius) (Acari: Amblyomminae) has been poorly investigated so far. Therefore, the effects of application of permethrin on engorged *D. reticulatus* females have been assessed, and the survival rate for the different developmental stages of the tick species in its non-parasitic phase of the life cycle was determined upon application of the pyrethroid.

**Methods:**

Each engorged *D. reticulatus* female, egg batch, unfed larvae (50 specimens), or unfed nymphs (10 specimens) were sprayed with 20 μl of 0.015625%- 0.250% solutions of a Coopex WP preparation, which corresponded to 0.78125- 12.5 μg of permethrin, respectively. The effect of permethrin in the preoviposition and oviposition periods was assessed based on the length of the egg development period, number of females capable of laying eggs, number and weight of eggs laid by the females, and indicators of female reproductive rate. Delayed effects of the action of the various permethrin doses were determined by quantitative and qualitative analysis of the course of embryonic development and larval hatch. The effect of permethrin on survival of the different tick stages was assessed at a temperature of 25°C and 90% RH 48 hours after application of permethrin to one to three day-old *D. reticulatus* eggs, seven to ten-day-old unfed larvae and nymphs.

**Results:**

The investigations demonstrated that at the doses applied permethrin decreased the reproductive rate of females, but only at the dose of 6.25 μg/1 specimen was the mean number of laid eggs drastically reduced, which may result in a decrease in tick abundance already in the first generation. The embryonic development of the eggs laid by the females treated with 6.25 and 12.5 μg of permethrin/1 specimen was highly disturbed. Irrespective of the permethrin dose applied, all eggs died within 48 hours after application. 100% of unfed *D. reticulatus* larvae and nymphs died within 48 hours after application of permethrin doses of 6.25 μg/50 specimens and 12.5 μg/10 specimens, respectively.

**Conclusions:**

Permethrin can be recommended for *D. reticulatus* control, particularly in the case of tick resistance to other chemical substances.

## Background

*Dermacentor reticulatus* has its geographic range in the Palaearctic, including Europe [1]. In Poland, the species inhabits areas along the border with Lithuania, Belarus, and Ukraine in the east and Slovakia in the south, although recently it has been found in other parts of the country as well [2].

The greatest economic and epidemiological importance of the *D. reticulatus* tick is associated with infestations of domestic and livestock animals, which can be thereby infected with pathogens. The species transmits tick-borne diseases, primarily canine babesiosis [3] and bacterial diseases [4-6], but also tick-borne encephalitis [4] and rickettsioses [7-9]. However, since it infests the same animals as *Ixodes ricinus* Linnaeus, the main vector of numerous pathogens in Europe, and occasionally attacks humans [7,10], *D. reticulatus* may play a certain role in circulation of human tick-borne diseases in nature. Although *D. reticulatus* with its short hypostome induces mechanical damage to the upper skin layers only, it causes extensive inflammatory lesions by saliva components secreted during feeding on the host [11].

Until recently, no integrated actions have been undertaken to control *D. reticulatus.* Investigations conducted on the species were mainly focused on the effect of acaricides on adult *D. reticulatus* stages during the parasitic stage on the host [12-15]. However, little is known about the effect of chemical substances on this tick species in the non-parasitic phase of its life cycle. In the present study, the effects of application of permethrin on various developmental stages of *D. reticulatus* and its potential practical use were assessed.

## Methods

### Tested acaricides

Permethrin (Coopex WP, AgrEvo Environmental Health Ltd., Great Britain) containing 25% of the active substance (sum of isomers: (1RS, 3RS)-(1RS, 3RS)-3-(2,2-dichlorovinyl)-2-dimethylcyclopropanecarboxylate 3-phenoxybenzyl; C_21_H_20_Cl_2_O_3_) was used in various concentrations obtained in a series of dilutions. The effect of 0.015625%, 0.03125%, 0.0625%, 0.125%, and 0.25% pyrethroid solutions was tested. 20 μl of the solutions used in each experiment contained 0.78125; 1.5625; 3.125; 6.25, and 12.5 μg of the active substance, respectively. Since the *D. reticulatus* females failed to lay eggs under the 0.5% solution, this concentration was not tested in the further steps of the investigations.

### Ticks maintenance

Adult stages of *D. reticulatus* were collected in the surroundings of Lublin (eastern Poland, 51°25′N) by flagging during the period of maximal spring tick activity (April- May) [16].

In the laboratory, the *D. reticulatus* ticks were maintained in rearing chambers at 5°C and 90% RH; prior to the beginning of the experiments, they were kept at room temperature of ca. 20°C and 90% RH for several hours. The ticks were fed on New Zealand albino rabbits (*Oryctolagus cuniculus*) at a temperature of ca. 20°C and ca. 50% humidity. 15 females and 5 males were placed on each rabbit. The course of feeding was assessed daily at the same time in order to collect engorged specimens immediately after detachment from rabbit’s skin. Engorged females were used for testing chemicals and establishing a laboratory culture in order to obtain various developmental stages of the species for further investigations on the effect of the chemicals on eggs, larvae, and nymphs.

The tick culture was performed in the laboratory at the Chair and Department of Biology and Parasitology, Medical University, Lublin, in accordance with the methodology, modified by a member of our research team (A. Buczek), in which conditions of culture of all *D. reticulatus* developmental stages were optimized. Larvae and nymphs (both unfed and engorged) in the non-parasitic stage were kept in rearing chambers at a temperature of 25°C and 90% humidity. The unengorged larvae and nymphs fed on rabbits.

### Test procedure

Two experiments were carried out. In the first experiment, the effect of permethrin on engorged *D. reticulatus* females and the consequences of the toxicity of the substance on the development of the ticks were assessed. Immediately after detachment, engorged females were weighed with an accuracy of 0.01 mg using a Radwag WPA 120/C/1 analytical balance. Next, each engorged female was placed on Whatman filter paper in a separate rearing chamber; the dorsal part of the body was sprayed with 20 μl of solutions with the tested concentrations of the active substance using micropipette with an accuracy of ± 0.5-2.0%. Females treated with the different permethrin doses were kept in the dark at 25°C and 90% RH before and during oviposition. The females were observed under a stereoscopic microscope every day, which allowed determination of the length of the preoviposition period. After oviposition, each female and egg batch were weighed; next, the eggs were transferred to a thermostat and they remained in the rearing chambers at 25°C and 90% RH until completion of embryogenesis. Concurrently, control experiments were performed, in which the same procedures were employed, but the females were treated with 20 μl of distilled water instead of the chemical substance.

The following parameters were determined: the preoviposition period (PP, the period between the end of feeding and the onset of oviposition expressed in days), egg lying frequency (ELF, the number of engorged females in the tested group capable of laying eggs, expressed in %); female postoviposition weight (FPW, weight of female body after completion of oviposition, in grams); female oviposition weight loss (FOWL, the percentage of weight loss during the oviposition period defined as the ratio of the difference between engorged female weight and female postoviposition weight/engorged female weight, in %); egg mass weight (EMW, the total weight of eggs laid by one female, in mg); egg conversion factor (ECF, indicating the weight of an engorged female used for production of eggs); and egg amount (EA, the total number of eggs laid by one female). The parameters of embryonic development assessed included hatching frequency (HF, the percentage of egg batches in the tested group with at least one hatched larva, expressed in %); the length of the embryogenesis period (EP, the period between the onset of oviposition and hatching of the first larva, in days); hatching success (HS, specifies the proportion of larvae hatched from eggs laid by one female, in %); as well as the proportion of dead eggs, dead embryos in embryogenesis stage I, II, and III, larvae with morphological anomalies, larvae with hatching disturbances, and normal larvae (in accordance with the criteria presented by Buczek et al. [17]).

The second experiment consisted in examination of survival of eggs, larvae, and nymphs upon application of the same permethrin concentrations as those used in the first experiment. One egg batch composed of one- to three-day-old eggs, 50 seven to ten-day-old unfed *D. reticulatus* larvae, and 10 seven to ten-day-old unfed nymphs originating from the laboratory colony, were placed in each rearing chamber; next, they were kept in the dark at 25°C and 90% RH. The eggs and the larvae and nymphs were sprayed with 20 μl of the tested permethrin solutions. In this part of experiment, 141,984 eggs (ten egg batches), 1800 unfed larvae, and 360 unfed nymphs were used in the experiment. After 48 hours, each experiment was viewed under a stereoscopic microscope to estimate the number of dead and live eggs or active *D. reticulatus* stages. The eggs, larvae, and nymphs from the control group were sprayed with 20 μl of distilled water.

### Statistical analysis

The calculations were done using the Statistica 5 PL program. The significance of the differences between the parameters of the non-parasitic stage of the ticks in the control group and the respective experimental groups treated with the specified concentrations of the chemical substance was estimated using the Mann-Whitney *U* test. The Kruskal-Wallis H test was employed for verification of the hypothesis of equality of the parameters for the respective concentrations of the acaricide. In both tests, the difference was regarded as statistically significant at p ≤ 0.05, and highly significant at p ≤ 0.01.

### Ethical approval

The study was carried out with the full approval of Commission for Animal Experiments.

## Results

Application of permethrin resulted in a decrease in the proportion of egg-laying females in the range of 83.3% at the lowest concentrations to 16.7% at the highest (control 100%) (Table 1). The increase in the permethrin concentration was accompanied by a statistically significant increase in the length of the preoviposition period. Upon application of the higher solution concentrations of 0.125 and 0.250% (permethrin doses 6.25 and 12.5 μg/1 specimen), there was a 5-fold increase to 11.33 ± 3.51 days and 10.50 ± 0.7 days, respectively, compared with the control (2.16 ± 0.64 days). The statistically significant differences in the preoviposition length in the groups of engorged females treated with the different permethrin doses were confirmed as well (test H = 46.26052, p = 0.0000). As a result of the toxic effect of permethrin doses of 0.78125-3.125 μg/1 specimen (0.01525-0.0625% solutions), the females laid a lower number of eggs (on average from 2165 to 2405 eggs) than in the control group (on average 3966.3 eggs). At the doses of 0.78125 - 3.125 μg/1 specimen, there were no considerable differences between the egg batches. In turn, application of permethrin at the doses of 6.25 and 12.5 μg/1 specimen (0.125 and 0.25% solutions), the number of eggs laid decreased ca. two- and three-fold, respectively, in comparison to the control. A high percentage of eggs that were dead at oviposition were found under these experimental conditions. The permethrin dose of 12.5 μg/1 specimen (0.25% solution) reduced the number of eggs in the batches to 837 on average. Since a low number of eggs were laid, utilisation of nutrient reserves by the females was also decreased, hence the indicator of female oviposition weight loss and the egg conversion factor were reduced, and the female postoviposition weight increased, compared with the control (Table 2). The egg mass weight was statistically significantly lower after the application of all the permethrin doses and ranged from 0.134 ± 0.041 g at the permethrin dose of 0.78125 μg/1 specimen (0.015625% solution) to 0.043 ± 0.048 g at application of 12.5 μg permethrin per female (0.250% solution) (control 0.221 ± 0.044 g) (Table 2). The egg mass weight differed between the experimental groups treated with the different concentrations of the active substance (test H = 38.12938, p = 0.0000).

**Table 1:** **Oviposition and embryonic development in *Dermacentor reticulatus *** **under the influence of different concentration of permethrin**

**Chemical**	**Concentration (%)**	**ELF (%)**	**EA**	**HF (%)**	**EP (days)**	**HS (%)**
Permethrin N = 59	0.01562	83.3	2165	90	19.66	90.25
	0.03125	66.7	2510.5	100	23.25	97.87
	0.0625	58.3	2405	100	21.74	86.5
	0.125	25.0	997	100	18.00	0.00
	0.25	16.7	837	100	23.00	0.84
**Control** N = 50		100	3966.3	100	12.2	98.15

N- number of ticks used in the experiment.

**Table 2:** **Eggs maturation and oviposition course in *Dermacentor reticulatus *** **females treated with different concentration of permethrin**

**Chemical**	**Concentration (%)**	**Preoviposition (days)**			**FPW (g)**			**FOWL (%)**			**EMW (g)**			**ECF**		
		**M**	**SD**	**p**	**M**	**SD**	**p**	**M**	**SD**	**p**	**M**	**SD**	**p**	**M**	**SD**	**p**
Permethrin N = 59	0.01562	5.60	2.366	0.0000	0.171	0.051	0.0124	56.504	14.363	0.0042	0.134	0.044	0.0001	0.337	0.100	0.0002
	0.03125	6.00	1.773	0.0000	0.149	0.058	0.3160	63.365	10.583	0.1236	0.120	0.041	0.0000	0.314	0.138	0.0016
	0.0625	6.57	1.718	0.0000	0.120	0.014	0.4612	71.664	3.161	0.4612	0.144	0.046	0.0011	0.337	0.106	0.0008
	0.125	11.33	3.512	0.0048	0.262	0.087	0.0070	39.852	22.861	0.0145	0.070	0.020	0.0048	0.160	0.048	0.0048
	0.250	10.50	0.707	0.0195	0.163	0.050	0.2129	56.926	2.775	0.0292	0.043	0.048	0.0195	0.135	0.161	0.0195
**Control** N = 50		2.16	0.648	x	0.127	0.023	x	69.547	5.801	x	0.221	0.044	x	0.531	0.119	x

N- number of ticks used in the experiment.

Due to the action of permethrin applied at the doses from 0.78125 to 3.125 μg/1 specimen (0.015625-0.0625% solutions), the period of the embryonic development of the *D. reticulatus* eggs was statistically significantly extended from 19.667 ± 6.819 to 23.25 ± 11.158 days, compared with the control (12.2 ± 0.610 days). Similar statistically significant differences in the duration of embryogenesis (test H = 29.78399, p = 0.00) were noted between the experimental groups treated with these doses (Table 1). Permethrin doses in the range from 0.78125 to 3.125 μg/female (0.015625-0.0625% solutions) induced inconsiderable disturbances in the embryonic development; hence, a large number of morphologically normal larvae hatched (Table 3). Application of the higher doses of 6.25 and 12.50 μg/1 specimen (0.125 and 0.250% solutions) yielded mortality of all eggs or inhibition of egg development in embryogenesis, disturbed larval hatch, and lack of normally developed larvae. Morphological anomalies in *D. reticulatus* larvae were rarely found. They affected the walking legs and consisted in fusion (heterosymely) or lack (oligomely) thereof.

**Table 3:** **The course of embryonic development in *Dermacentor reticulatus *** **under the influence of permethrin**

	**Permethrin concentration (%)**					**Control N = 23,797**
	**0.01562 N = 12, 990**	**0.03125 N = 15,063**	**0.0625 N = 14,430**	**0.125 N = 5,982**	**0.250 N = 5,022**	
Normal larvae (%)	90.25	97.87	86.46	0.00	0.84	98.15
Larvae with developmental anomalies (%)	0.00	0.00	0.04	0.00	0.00	0.00
Larvae with hatching disturbances (%)	0.83	0.15	0.68	0.00	72.88	0.13
Dead eggs (%)	1.02	0.20	2.14	100	2.75	0.07
Inhibition in embryogenesis (%)	7.90	1.78	10.67	0.00	23.54	1.66
In the I stage (%)	68.42	10.38	74.62	0.00	37.06	30.06
In the II stage (%)	26.32	24.53	17.93	0.00	31.98	57.48
In the III stage (%)	5.26	65.09	7.45	0.00	30.96	12.47

N- number of *D. reticulatus* eggs included in six egg batches analyzed to determine the course of embryonic development and larval hatch under the influence of different permethrin concentrations.

At the temperature of 25°C and 90% RH, all *D. reticulatus* eggs died after 48 hours, irrespective of the permethrin concentration applied (Table 4). Under these conditions, the survival rate for unfed larvae treated with the 0.01563% acaricide solution (0.78125 μg permethrin dose /50 specimens) was only 6.7%. The higher concentrations of the pyrethroid resulted in mortality of all larvae. After 48 hours, a larger number of unfed nymphs than larvae survived. At the lowest tested concentrations of permethrin, survival of unfed nymphs was significantly lower (20%) than in the control (93.3%) (Table 4). At the dose of 6.25 μg/1 specimen (0.125% solution), permethrin caused 100% mortality of unfed *D. reticulatus* nymphs.

**Table 4:** **Survival of eggs, larvae and nymphs of *Dermacentor reticulatus *** **48 hours post application of permethrin**

**Acaricide**	**Acaricide concentration (%)**	**Eggs (%)**	**Unfed larvae (%)**	**Unfed nymphs (%)**
		**N**^**1**^ **= 141 984**	**N**^**2**^ **= 1 800**	**N**^**3**^ **= 360**
Permethrin	0.01563	0.00	6.7	20.0
	0.03125	0.00	0.00	6.7
	0.0625	0.00	0.00	6.7
	0.125	0.00	0.00	0.00
	0.250	0.00	0.00	0.00
Control		93.3	98.1	96.7

N^1^-number of *D. reticulatus* eggs used to determine the survival under the influence of different permethrin concentrations; N^2^- number of *D. reticulatus* larvae used to determine the survival under the influence of different permethrin concentrations; N^3^- number of *D. reticulatus* nymphs used to determine the survival under the influence of different permethrin concentrations.

## Discussion

Previous investigations showed the effectiveness of permethrin [18-22] and combinations of permethrin with other acaricides [23-26] in the control of several tick species, including *D. reticulatus*. Our present results have confirmed that permethrin decreases the reproductive rate of *D. reticulatus* females, with a drastic decline at the higher doses in the range of 6.25-12.5 μg/1 specimen (0.125 and 0.250% solutions). The application of the acaricide on engorged females significantly reduced the number and weight of laid eggs, probably by inhibition of the development of part of the oocyte pool in the ovaries of the experimental females, which we did not investigate.

Our observations indicate that application of permethrin changed the dynamics of oocyte development, which prolonged the preovipositon period. The length of the preovipositon period in *D. reticulatus* was correlated with the increasing doses of permethrin applied to engorged *D. reticulatus* females. These results are in agreement with the studies conducted by other authors [27], who reported morphological changes in oocytes of semi-engorged *Rhipicephalus sanguineus* Latreille females induced by permethrin, including emergence of large vacuolated cytoplasm regions, lower amounts of yolk, and reduced size of oocytes. In our experiments, application of the higher permethrin doses reduced the number of *D. reticulatus* eggs; additionally, some eggs were morphologically changed and incapable of further development at the time of leaving the female genital tract. Such eggs were strongly shrunken and devoid of fluid. The reduced reproductive rate in *D. reticulatus* females suggests considerable permethrin-induced disturbances during the vitellogenic phases of oocyte development. In ticks, the vitellogenic phase begins with appearance of the first yolk granules and ends before ovulation [28]. In females of most species of ixodid ticks, blood meal ingestion and copulation are indispensable for completion of vitellogenesis. Yolk proteins are converted from host blood proteins by gut cells and/or fat-body cells and released into the hemolymph [29]. Roma et al. [27] showed that increased permethrin concentrations caused a decline in the number of yolk granules in oocytes IV and V in *Rh. sanguineus*, compared with the control. Based on examinations of ovaries of *Amblyomma hebraeum* Koch females treated with another synthetic pyrethroid, i.e. cypermethrin, Friesen and Kaufman [30] reported a decrease in the number of vitellin elements in oocytes and reduced synthesis of ecdisteroids, which exert an effect on production and release into the hemolymph of vitellogenins, the major yolk proteins.

Decreased reproductive rate of tick females is manifested in a smaller number of offspring and, consequently, decreased tick population abundance in a given area. It is also highly important for the transovarial pathogen transmission, which is an extraordinary ability of ticks as they can thus transmit viruses, bacteria, rickettsiae, and protozoa. The eggshell protects oocytes from pathogen penetration. It has been demonstrated that only young oocytes in *Dermacentor andersoni* Stiles can be infected by *Rickettsia*[31]. Similarly, *Coxiella burnetii* penetrates young oocytes in *Hyalomma asiaticum* Schulze & Schlottke [28], and *Babesia duttoni* infects young oocytes in *Haemaphysalis punctata* Canestrini and Fanzago [32]. In turn, *Rickettsia rickettsii* was detected in oocytes at various development stages (I, II, III, IV) in *Rh. sanguineus*[33]. Probably, abnormal development of the eggshell induced by chemical compounds, e.g. pyrethroids, facilitates penetration of pathogens in the various stages of oocyte development. Our investigations of the effect of other pyrethroids (cypermethrin, alphacypermethrin, and deltamethrin) on engorged *D. reticulatus*[17] and *I. ricinus* (unpublished data) females demonstrated a more pronounced effect of the compounds during this stage of the life cycle of these two tick species. The comparison of the results obtained in our investigations indicates that among the pyrethroids tested permethrin exerts a moderate toxic effect on *D. reticulatus*.

The high mortality of eggs laid by females treated with the highest concentrations of permethrin and embryo mortality during embryogenesis may be related not only to the decrease in the amounts of stored yolk proteins required for further development, but also to disturbances in the egg-shell synthesis, which begins in stage III and ends in stage IV of oocyte growth before ovulation [28]. To our knowledge, no research has been done into structural changes of the tick egg-shell induced by chemical substances. In normal tick physiology, the egg-shell and lipid-rich glandular secretion covering eggs during oviposition protect eggs against the harmful effect of environmental factors.

Oocytes in the ovaries develop asynchronously [27,28,30,34]. In turn, absorbed pyrethroids are metabolised in tick organism, and their toxic effect probably changes. This may explain the fact that at the 0.125% concentration of the solutions all *D. reticulatus* eggs died within 1-3 days after oviposition, while at the higher 0.25% concentration the development of some eggs was inhibited at the later stages of embryogenesis, and larval hatch was inhibited as well.

Permethrin induced fewer disturbances in the course of the embryonic development in *D. reticulatus* than the other pyrethroid tested in our previous research [17]. In the present study, we observed fewer dead embryos and fewer cases of inhibited larval hatch than those induced by deltamethrin and cypermethrin, and fewer larvae with morphological anomalies than those produced by the action of deltamethrin [17]. Our investigations demonstrate that the delayed effects of permethrin toxicity to ticks are considerably less pronounced than the effects induced by other pyrethroids, e.g. cypermethrin, alphacypermethrin, and deltamethrin [17], (Buczek et al. unpublished). Developmental disorders in ticks were reported to be caused by other chemical substances [35,36] and thermal shock [37] as well as adverse humidity conditions [38] during embryogenesis.

In our study, permethrin caused high mortality of eggs and the active developmental stages of *D. reticulatus*. The effectiveness of the acaricidal effect of permethrin was observed in various developmental stages in other tick species. According to Burridge et al. [20], among the four acaricides tested (cyfluthrin, pyrethrins, amitraz, permethrin), permethrin and cyfluthrin were reported to be the most efficient substances for control of adult stages of *Amblyomma cajennense* (Fabricius), *A. maculatum* Koch, and *A. americanum* Linnaeus in the USA. In the case of permethrin, such an effect was achieved in *A. americanum*, regardless of whether it was applied directly on nymphs [39] or hosts infested by adult stages [40]. In turn, permethrin appeared to be less effective among 15 pyrethroids tested for their acaricidal effect on unengorged *Ixodes persulcatus* Schulze females. LD_50_ in μg g^-1^ for permethrin was 0.43 ± 0.08, while in the case of deltamethrin, i.e. the most efficient pyrethroid, it reached 0.034 ± 0.006 [18]. Comparison of our results of the research on the effect of various permethrin doses on *D. reticulatus* with studies carried out on other tick species is difficult due to the different methodologies employed. Acaricidal activity is also exhibited by other pyrethroids, e.g. flumethrin [41-43].

The study of Uspensky and Ioffe-Uspensky [44] showed that larger tick species (*Dermacentor silvarum* Olenev, *Rhipicephalus turanicus* Pomerantsev, Matikashivili & Lototsky, and *Hyalomma asiaticum* Schulze & Schlottke) are more reflactory to acaricides than small-sized ticks (*I. ricinus, I. persulcatus*, *Haemaphysalis concinna* Koch, and *Rh. sanguineus*), which results in more prolonged duration of poisoning development.

## Conclusions

Reduction of the high reproductive rate of ticks by application of chemical substances is a key element in tick control strategies, since it decreases the abundance of subsequent generations. Despite its modest effect in comparison to that of other pyrethroids, the use of permethrin for *D. reticulatus* control is better to consider as a safer acaricide for the environment.

## Competing interests

The authors declare that they have no competing interests.

## Authors’ contributions

AB designed the study, contributed to analysis and interpretation of data, drafted the initial manuscript, reviewed comments by co-authors. KB contributed to interpretation of data and to the statistical design and analysis plan, participated in writing of the manuscript. PK conducted statistical analyses contributed to acquisition of data. All authors read and approved the final manuscript.
